# Spectrum Analysis of Gravity Waves Based on Sensors Mounted on a New Round-Trip Airborne Flat-Floating Sounding System

**DOI:** 10.3390/s20072123

**Published:** 2020-04-09

**Authors:** Yang He, Zheng Sheng, Jie Zhang, Mingyuan He, Shudao Zhou

**Affiliations:** 1 College of Meteorology and Oceanography, National University of Defense Technology, Changsha 410073, China; heyang12357@sina.com (Y.H.); jaycheung8@sina.com (J.Z.); hmy008@sina.com (M.H.); zhousd70131@sina.com (S.Z.); 2 Collaborative Innovation Center on Forecast and Evaluation of Meteorological Disasters, Nanjing University of Information Science and Technology, Nanjing 210094, China

**Keywords:** physical sensors, gravity wave spectrum, detection technology, radiosonde

## Abstract

In this study, sensors mounted on a new type of round-trip airborne flat-floating sounding system (RTAFSS) were used to obtain the observation data of the three stages of "rising, flat-floating and falling". This new sounding method has a good application prospect. We performed spectrum analysis on the normalized temperature fluctuation, and the vertical wavenumber spectrum from the rising and falling stages and the horizontal wavenumber spectrum from the flat-floating stage were obtained. This is the first time the complete gravity wave spectrum characteristics were obtained from three consecutive stages: rising, flat-floating and falling. The results show that the gravity wave spectrum of the three stages can be well obtained by RTAFSS. For the horizontal wavenumber spectrum, the spectral slope is basically around −2, and the difference in the spectral structure of the horizontal wave number spectrum may be due to the intermittent turbulent activity and the variable intensity of the gravitational wave during its propagation. This study aims to make experimental exploration of the spectrum characteristics of gravity waves by this new type of observation data. It is expected to reveal the spectrum characteristics of horizontal wavenumber in the stratosphere region of China, providing a theoretical basis for spectrum analysis in a wider space–time range after further network observation of RTAFSS.

## 1. Introduction

Gravity wave is the main source of mesoscale fluctuation in the stably stratified atmosphere, with upward propagation of gravity wave, density decreases exponentially with height, leading to the increase of gravity wave amplitude. Gravity wave follows the evolution process including generation, propagation, saturation and breaking, continuously dissipating energy and making an important contribution to momentum budgets and turbulence at all altitudes. The fluctuation profiles of atmospheric wind field, temperature field, density field and other meteorological tracers contain the disturbance information of various scales, and the fluctuation profiles based on the detected data can extract the characteristics of the gravity wave spectrum, from which a series of gravity wave spectrum theories are developed. Because the spectrum characteristics of gravity waves are significantly affected by the Doppler effect generated by the mean background wind field [1], and methods including rocket detection [2], mesosphere-stratosphere-troposphere (MST) radar and middle-upper atmosphere (MU) radar measurements [3,4], lidar measurements, balloon observations [5,6,7] and other ground-based detection can only obtain vertical wavenumber spectrum. Therefore, many studies have focused on the vertical wavenumber spectrum of gravity waves to investigate the spectrum theory. A variety of gravity wave saturation models have been put forward, including linear instability [8,9], the nonlinear wave–wave interaction [10], Doppler extension [11,12] and saturated cascade similarity theory [13]. The concept of "universal" spectrum further proved that, in the troposphere and stratosphere, the vertical wavenumber spectrum in the saturated region has similar spectrum power and shape.

The theoretical model of horizontal wavenumber spectrum is also proposed on the premise that the gravity waves follow the polarization and dissipation relationship and that the wavenumber and frequency can be separated [12,14]. Due to the limited detection means, the observation of horizontal wavenumber spectrum of gravity wave is much less than that of vertical wavenumber spectrum. At present, the horizontal structure of gravity waves is mainly obtained through data samples collected by instruments mounted on detection aircraft [15,16,17]. However, aircraft detection is limited by time and space coverage. Wu applied spectrum analysis to the Microwave Limb Sounder (MLS) limb-tracking radiances and obtained the seasonal characteristics of the horizontal wavenumber spectrum [18,19]. The research on horizontal wavenumber spectrum is still worthy of in-depth study; the horizontal spectrum characteristics obtained through detection can especially provide important reference value for the verification and improvement of spectrum theory, which is currently lacking due to the limited detection methods.

Based on the data obtained by sensors mounted on RTAFSS, the vertical spectrum characteristics of gravity waves are analyzed by using the data from rising and falling stages, and the horizontal wavenumber spectrum characteristics are analyzed using the data from flat-floating stage. Long-term, high-frequency continuous direct observation of balloons in the stratosphere fills a gap in this aspect of China’s meteorological observations. The United States Stanford Space Program has attempted to develop a high-altitude balloon system based on latex balloons to achieve long-term stratospheric flight. However, there is currently no internationally developed three-stage sounding observation that can meet the whole process of "rising, flat-floating and falling". This paper applies spectrum analysis to the three-stage dataset of the RTAFSS for the first time. The application of this type of sounding data on the study of the gravity wave spectrum was explored, and the airborne direct observation of the horizontal wavenumber spectrum of gravity waves was obtained based on long-time interval of balloon flat-floating for the first time.

## 2. Data and Methods 

### 2.1. System Introduction and Principle

**Figure 1:**
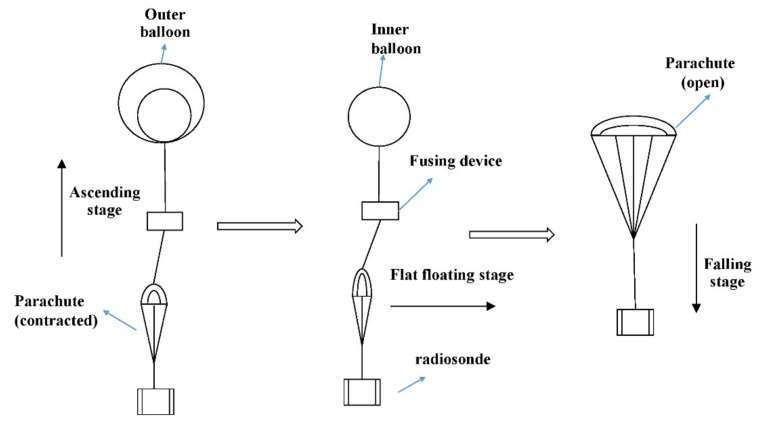
Schematic diagram of round-trip airborne flat-floating sounding system (RTAFSS).

The data used in this paper are from sensors mounted on the round-trip airborne flat-floating sounding system (RTAFSS). This system is composed of load, carrier and ground receiving system, and it can realize the three-stage detection process: “rising, flat-floating and falling”. The diagram of the system is shown in Figure 1. The load includes radiosonde and fusing device. The radiosonde consists of a Beidou navigation sensor, meteorological sensor module (sampling frequency 0.5 Hz), transmission modulation module, power amplifier, battery and corresponding peripheral circuits. This type of Beidou chip supports meteorological-data-processing functions, including functions such as data acquisition, calculation and storage, and it can control the data exchange between the transmitting device and the ground. The integrated radiosonde is characterized by low power consumption (<100 mW), miniaturization (<100 g), long-distance communication (>200 km), etc. The fusing device is used to separate the inner balloon from the parachute. The outer balloon, the inner balloon and the parachute are respectively used as the carriers for the rising, flat-floating and falling stages. The outer balloon provides the rising power to realize the conventional rising, and it explodes at a predetermined height. Then the inner balloon realizes the long-time flat-floating (>4 h); finally, the controlled dropping is realized by the fusing device and encrypted observation is achieved through the parachute carrying the radiosonde. The sensor module consists of three parts: (1) a negative temperature coefficient (NTC) thermistor sensor for temperature measurement, with the measurement range from −90 to 50 °C (ΔT≤0.3 °C); (2) a piezoresistive sensor for air pressure measurement, with the advantages of large compensation dynamic range and high accuracy; and (3) a humidity-sensitive capacitance sensor, which has the advantages of having a wide humidity measurement range, a fast response speed, a small volume, high sensitivity, a small hysteresis coefficient, a small hysteresis loop and a small temperature coefficient (ΔRH <± 1.5% RH).

During the ascent of the balloon, the balloon is affected by buoyancy, air resistance and gravity in the vertical direction, while there is no external force acting in the horizontal direction. It is approximately moving with the air mass. The movement mode in the ascent stage is as follows [20]:

(1) $$m\frac{dw}{dt}=\rho Vg-mg-\frac{\pi }{8}\rho C_{p}D^{2}w^{2}$$

where m is the total mass of the system, g is the acceleration of gravity, w is the ascending speed, V is the outer ball volume, ρ is the air density, $C_{p}$ is the drag coefficient and D is the outer ball diameter.

In the flat-floating stage, the system makes an approximate horizontal movement, and the vertical force is approximately balanced, with slight fluctuation. The adaptive flat-floating process is achieved by controlling the appropriate net lift force on the ground. The movement mode in the flat-floating stage is calculated as follows:

(2) $$F_{up}=\rho_{0}g\frac{V_{h}P_{h}T_{0}}{T_{h}P_{0}}-mg$$

(3) $$V_{h}=mg/\rho_{h}$$

where $P_{h}$, $T_{h}$ and $\rho_{h}$ are respectively the air pressure, temperature and density of the balloon at height (h); and $T_{0}$, $P_{0}$ and $\rho_{0}$ are the air pressure, temperature and density of the balloon when it is on the ground. When the balloon begins to fall, the inner ball and the parachute are separated by the fusing device, the parachute is fully opened and the radiosonde is falling from the low-density area to the high-density area. At this time, the parachute needs to consider the forces in three directions, and the movement mode in the falling stage is as follows:

(4) $$m\frac{dV_{ux}}{dt}=-m_{au}\frac{dV_{x}}{dt}-\frac{\rho SV_{r}\left(C_{d}V_{x}+C_{l}\left(kV_{y}-jV_{z}\right)\right)}{2}$$

(5) $$m\frac{dV_{uy}}{dt}=-m_{au}\frac{dV_{y}}{dt}-\frac{\rho SV_{r}\left(C_{d}V_{y}+C_{l}\left(-kV_{x}+iV_{z}\right)\right)}{2}$$

(6) $$m\frac{dV_{uz}}{dt}=m_{f}g-mg-m_{au}\frac{dV_{z}}{dt}-\rho SV_{r}\left(C_{d}V_{z}+C_{l}\left(jV_{x}-iV_{z}\right)\right)/2$$

where m is the mass of the system, $m_{au}$ is the additional mass due to the accelerated movement of the parachute, $m_{f}$ is the mass of air exhausted by the system, ρ is the air density and S is the area when the parachute is fully open. $V_{x}$, $V_{y}$ and $V_{z}$ are velocity of the radiosonde relative to air, $V_{r}=\sqrt{V_{x}{}^{2}+V_{y}{}^{2}+V_{z}{}^{2}}$. $V_{uy}$, $V_{ux}$ and $V_{uz}$ are the speed of the radiosonde itself; $C_{d}$ is the drag coefficient; and $C_{l}$ is the lift coefficient, respectively. Moreover, i, j and k represent the cosine of the three direction vectors.

### 2.2. Experiment Details and Data Processing

**Figure 2:**
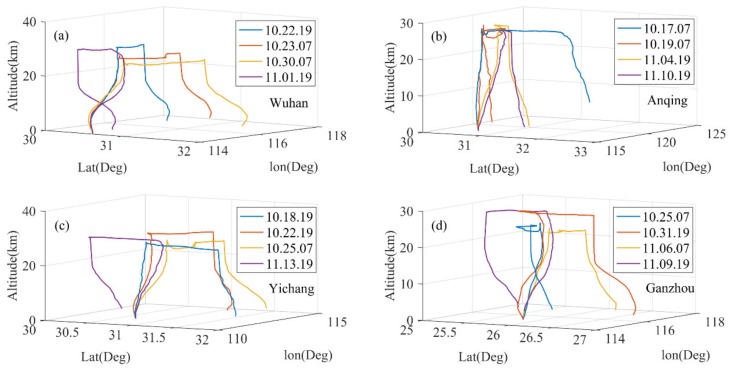
The complete detection trajectory of the sounding system, including three stages of “rising, flat-floating and falling”, released in (**a**) Wuhan, (**b**) Anqing, (**c**) Yichang and (**d**) Ganzhou.

The data used in this paper are a total of 16 groups of complete observation data, including the three stages of "rising, flat-floating and falling" released from Wuhan, Anqing, Yichang and Ganzhou in 2018. The trajectory of each detection process is shown in Figure 2. Since the system has no power to control the direction during the flat-floating process, the balloon moves with the air mass at the corresponding height, and the obvious inconsistent flat-floating trajectory can be seen in the figure. For the balloons released at the same station, the drop points are also quite different. For each complete detection process of the RTAFSS, the specific time, altitude and average vertical speed of the three stages of "rising, flat-floating and falling" are shown in Table 1. The rising time of the balloon ranges from 1.2 to 1.9 h, and the flat-floating time is more than four hours. The longest flat-floating time is 8.2 hours for the detection system released in Anqing, on October 17, 2018. The duration of the descending phase is also between 1 and 2 hours, except for the slower falling speed on October 17 and October 19 in Anqing, where the duration lasted more than three hours. Except for the data from Anqing on October 22, the up-and-down height interval of the flat-floating stage is within 3 km, and the minimum interval is only 400 m.

**Figure 3:**
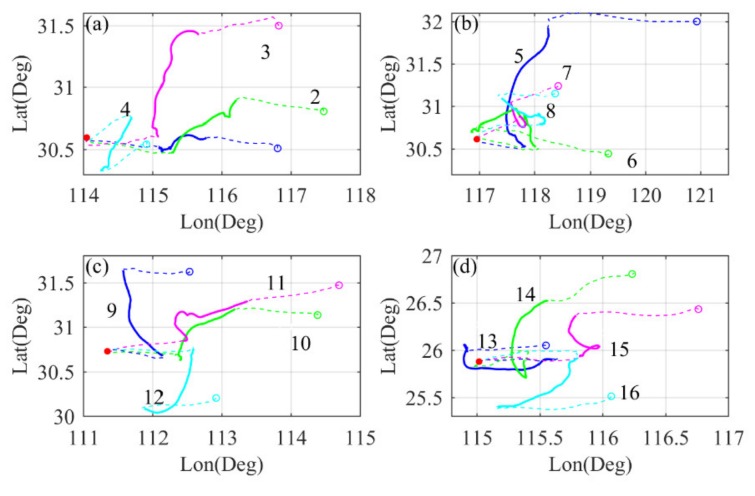
A top view of the latitude and longitude motion trajectories of RTAFSS in (**a**) Wuhan, (**b**) Anqing, (**c**) Yichang and (**d**) Ganzhou. The red solid points represent the station location; the blue, green, purple and cyan hollow points represent the last data reception position of the radiosonde. The two dashed lines represent the ascending and descending trajectories, respectively, and the solid line segments represent the flat-floating trajectory.

Figure 3 shows the top view of the complete trajectory of the three phases. Figure 3a–d represents the trajectories of RTAFSS releases in Wuhan, Anqing, Yichang and Ganzhou, respectively. The solid red dots represent the locations of the release point, and the blue, cyan, green and purple hollow dots represent the positions at which the radiosonde ends receive data. The dashed lines at the beginning and the end represent the trajectories of the rising and falling stages, and the solid lines represent the trajectories of the flat-floating segment. The number marked on each track corresponds to the release sequence in Table 1. The vertical velocities in the three stages of "rising, flat-floating and falling" change with the specific weather conditions at that time, so the resolution of different detection data is not the same. Before performing the spectrum analysis, we divide each set of data into three stages and perform uniform interpolation separately. The data in rising and falling stages are uniformly interpolated for height, and the vertical step length is 12 m. For the flat-floating stage, because the horizontal trajectory is very irregular, we need to perform data interpolation and resampling in two directions of meridian and zonal. Since the satellite navigation system can obtain latitude, longitude and altitude data, we calculate the real-time X, Y and Z position coordinates of the radiosonde in the topocentric terrestrial coordinate system. The position coordinates ($X_{0},Y_{0},Z_{0}$) of the release point at the station in the geocentric coordinate system should be calculated first:

(7) $$\left\{\begin{array}{l} X_{0}=(N+h_{0})\cos\varphi \cos\lambda \\ Y_{0}=(N+h_{0})\cos\varphi \sin\lambda \\ Z_{0}=[N(1-e^{2})+h_{0}]\sin\varphi \end{array}\right.$$

where λ and φ are the latitude and longitude (°) of the station, and $h_{0}$ is the altitude (m) of the station; N is the curvature radius (m) of the earth ellipsoid at the station location, $N=\frac{a}{\sqrt{1-e^{2}\sin^{2}\varphi }}$; e is the eccentricity of the earth ellipsoid, $e^{2}=\frac{a^{2}-b^{2}}{a^{2}}$; a is the long semi-axial length of an ellipsoid, a=6378137.0 m; and b is the short semi-axial length of an ellipsoid, b=6356752.3141 m.

The coordinate position ($X_{i},Y_{i},Z_{i}$) of the radiosonde at the corresponding ith measurement in the topocentric terrestrial coordinate system is calculated as follows:

(8) $$\left\{\begin{array}{l} X_{i}=\left(X_{0}\cos\left(\lambda_{i}\right)+Y_{0}\sin\left(\lambda_{i}\right)\sin\left(\varphi_{i}\right)-Z_{0}\cos\left(\varphi_{i}\right)\right)\\ Y_{i}=X_{0}\sin\left(\lambda_{i}\right)-Y_{0}\cos\left(\varphi_{i}\right)\\ Z_{i}=N+h_{0}-\left(X_{0}\cos\left(\lambda_{i}\right)+Y_{0}\sin\left(\lambda_{i}\right)\right)\cos\left(\varphi_{i}\right)-Z_{0}\sin\left(\varphi_{i}\right) \end{array}\right.$$

In this way, we obtained the coordinate position of the airborne detection system at each moment, where $\varphi_{i}$ and $\lambda_{i}$ are the latitude and longitude of the station (°). The X axis of the topocentric terrestrial coordinate system points to the north; the Y axis points to the east; and the Z axis passes through the origin and is perpendicular to the X and Y axes to the zenith. The straight-line distance from the radiosonde to the station is slope distance, L:

(9) $$L=\sqrt{{\left(X_{i}-X_{0}\right)}^{2}+{\left(Y_{i}-Y_{0}\right)}^{2}+{\left(Z_{i}-Z_{0}\right)}^{2}}$$

For the data segment from the flat-floating stage, we interpolate according to the X and Y directions. Since the horizontal step length in the X and Y directions of different profiles are quite different, the step length cannot be unified in the horizontal direction. Therefore, interpolation is performed according to the average horizontal step length of each group of data in the X and Y directions.

### 2.3. Gravity Wave Analysis

In this study, a Savitzky–Gola filter was selected, and a sliding polynomial fit was used to extract the gravity wave background profile. The filter coefficient was calculated by unweighted linear least squares regression and cubic polynomials, and the window width was selected to be 10 km, as it can reduce the errors generated by conventional fitting methods [21]. Perturbation residual is obtained by subtracting the background profile from the original data. Here we use the normalized perturbation ${\hat{T}}_{i}=T_{i}{}^{\prime }/T_{i0}$, where ${T^{\prime }}_{i}$ is the perturbation residual and $T_{i0}$ is the background profile(1≤i≤K, where K is the total data bins). The normalized data is pre-whited to reduce spectrum leakage: $P_{i}={\overset{⌢}{T}}_{i+1}-{\overset{⌢}{T}}_{i}$. Then, the fast Fourier transform is used for spectral transformation:

(10) $$F(k_{n})=\sum_{j=0}^{K-1}P_{j+1}\exp(-2\pi i\frac{nj}{K})$$

where $k_{n}=(n/K\Delta x)$, n = 1, …, K; $k_{n}$ is the wavenumber; and Δx is the step length. Unilateral power spectrum density (PSD) is calculated as follows:

(11) $$\Phi \left(k_{n}\right)=\left[\frac{2\Delta x}{K}\right]{\left|F\left(k_{n}\right)\right|}^{2}$$

Then the Hanning window is used to smooth the power spectrum. The smoothed power spectrum also needs to be recovered from the pre-whitening process, to compensate for the effects of the difference and cosine taper windows [22]. Considering that the high-wavenumber region of the temperature spectrum is attenuated too much above 20 km, the effect of the sensor lag on the temperature spectrum needs to be corrected [6]. The specific calculation process can refer to [22,23]. For the vertical wavenumber spectrum, the saturated gravity wave model used here is as follows [5,9]:

(12) $$F_{T^{\prime }/T_{0}}=\frac{1}{4\pi^{2}}\frac{N^{4}}{10g^{2}k_{n}{}^{3}}$$

where g is the gravitational acceleration, N is the Brunt–Vaisala frequency and the vertical wavenumber is $k_{n}$ in cycles per meter.

## 3. Results

### 3.1. Research of Detected Data

**Figure 4:**
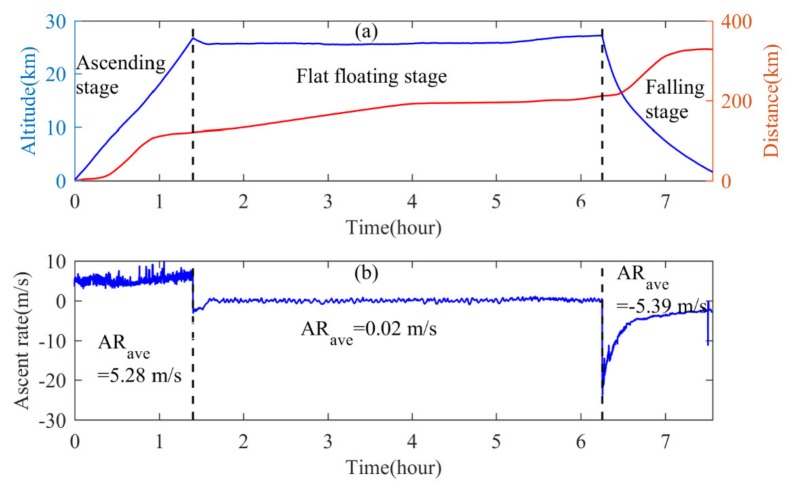
(**a**) Variation in altitude (blue curve), slope distance (red curve) with time and (**b**) variation in vertical ascend rate with time. Two vertical dashed lines are used to distinguish the three stages of "rising, flat-floating and falling".

We now take the data from Wuhan on October 23 as an example to analyze the data characteristics of RTAFSS. Figure 4a shows the variation in altitude and slope distance of the sounding system over time. The solid blue curve represents the altitude, and the solid red curve represents the slope distance. Figure 4b shows the variation of the vertical speed with time, where upward is positive. The ascending movement is approximately uniformly with time, and the average vertical speed is 5.28 m/s. After the outer ball explodes, there is a short sudden drop in height with time, which is a process of finely controlling the residual amount. Afterward, the atmospheric buoyancy–gravity balance is achieved at a predetermined height, leading to a stable long-time flat-floating. The average vertical speed during flat-floating is 0.02 m/s. The variation of altitude with time is basically small. When the inner balloon and the parachute begin to separate, the descent phase starts, and the radiosonde starts free-falling. After the parachute is opened, the initial fall speed is large, and the maximum fall speed can reach 25 m/s. Under the resistance of the parachute, the falling speed begins to gradually decrease, and the system moves from the low-density area to the high-density area. Until the radiosonde drops near the lower stratosphere, the falling speed is approximately the same as the rising speed, and the descending state of the system tended to be stable. The average falling speed of the entire descending process is 5.39 m/s. Therefore, during the falling stage, first the height decreases quickly with time and then becomes slower. At the beginning of the falling stage, it takes only 638 s to descend from 27 to 18 km. In the flat-floating stage, the balloon moves with the air mass, and the height is almost constant, so the slope distance increases very slowly, while in the rising and falling stages, the height and horizontal distance increase with time, the slope distance increases significantly and there is a sudden increase trend of slope distance around 10 km, which is caused by the rapid increase of the horizontal distance when the detection system passing through the jet stream area.

**Figure 5:**
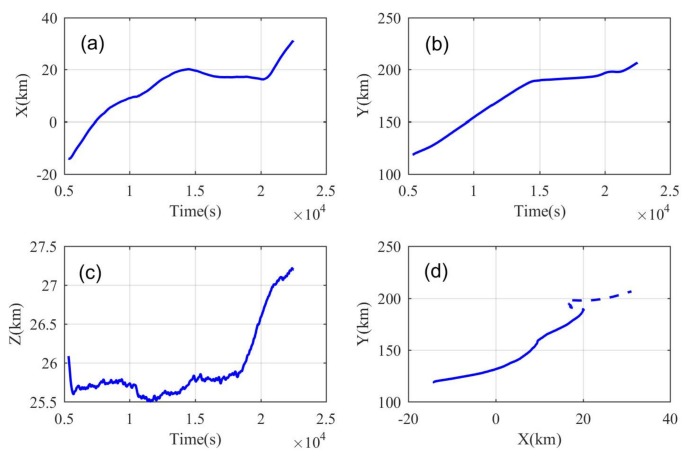
The variation of (**a**) X coordinate, (**b**) Y coordinate and (**c**) Z coordinate with time; and (**d**) the trajectory of the airborne radiosonde in the XOY plane, where the solid line is the data segment selected for spectrum analysis.

We selected the data of the flat-floating stage separately and calculated the topocentric terrestrial coordinates, the variation of X, Y and Z coordinates, and horizontal two-dimensional trajectories of the radiosonde with time are shown in Figure 5a–d. The X direction is the north direction, the Y direction is the east direction and the origin of the coordinates $\left(X_{0},Y_{0},Z_{0}\right)$ is the station location. During the flat-floating stage, the X coordinate moved from −14 to 31 km and moved by 45 km; the Y coordinate moved from 120 to 206 km and moved by 86 km; and the Z coordinate stabilized within the height range of 25.5–27.2 km. The zonal movement distance is greater than the meridional movement distance, indicating that, even in this corresponding height interval in the stratosphere, the zonal wind is greater than the meridional wind. The synthesized horizontal motion trajectory is shown in Figure 5d. If we want to extract the characteristics of the gravity wave spectrum from the data obtained by the airborne flat-floating sounding system, we need to first select the appropriate data segment. Here our selection principle is as follows: The data segment for horizontal wavenumber spectrum analysis must satisfy that the X and Y coordinates are monotonic. For example, the solid line part in Figure 5d represents the data of the horizontal segment selected for the spectrum analysis by this sounding data. Then, according to the X and Y directions, the meridional wavenumber spectrum and the zonal wavenumber spectrum are calculated. The other datasets are processed in the same way.

**Figure 6:**
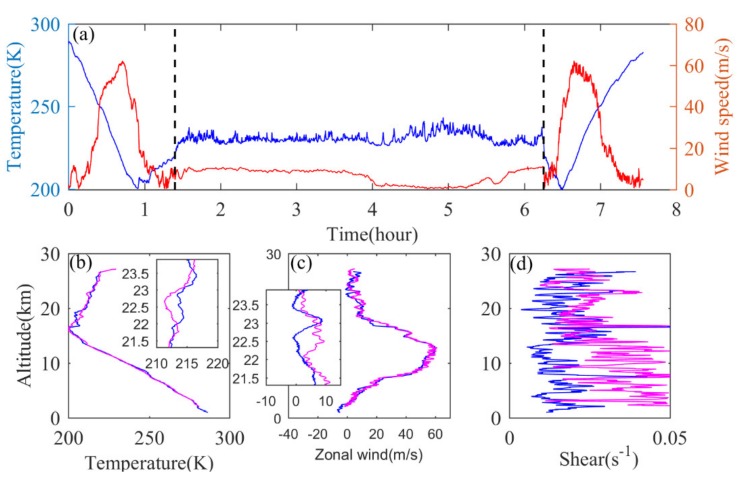
(**a**) Variations in temperature (blue curve) and wind speed (red curve) over time; vertical profiles of (**b**) temperature, (**c**) zonal wind, (**d**) vertical wind shear in the ascent (blue curve) and descending phases (purple curve).

Figure 6a shows the variation trend of measured temperature and wind speed with time, while Figure 6b–d shows the temperature, zonal wind and wind shear profile in the rising and falling stages, respectively. The overall trend of temperature profile and wind speed profile in the rising and falling stages is consistent, which is shown as approximate symmetry in Figure 6a. In order to compare the temperature and wind speed profile in the rising and falling stages in a more detailed way, the data segment range from 21.3 to 24 km is selected individually, and the profiles of the corresponding part is enlarged. It can be seen that, for the temperature profile, the data are directly measured by the temperature sensor, and there is little difference in the perturbation magnitude between the ascending and descending segments. The wind speed and direction are calculated by the positioning coordinates of satellite navigation. For the wind profile, there is obvious jagged jitter in the descending section. Considering that in the rising stage the outer balloon carries the radiosonde rising slowly and steadily, while during the falling stage, the radiosonde falling in the stratosphere is significantly faster than that in the troposphere, so it will be difficult to capture the fine structure of the wind speed measurement and affect the quality of the calculated wind speed data. At the same time, down to about 10 km, the slope distance is too large, the detection distance is too far and the ground station cannot track the signal effectively due to the low elevation angle occlusion, which will cause the lack of data measurement; this needs to be solved through post-interpolation and quality control. The above reasons cause the wind speed profile in the descending section to be relatively smooth. Compared with the wind speed profile in the ascending section, a large number of fine disturbance structures are missing. In Figure 6d, it can also be seen that the wind shear in the descending stage is obviously larger, especially in the troposphere. The abnormal wind shear indicates the discontinuity of wind speed change and the obviously larger error of wind calculated in the descending stage. In view of the above situation, in this paper, we mainly carried out spectrum analysis on the temperature perturbations in the three stages: “rising, flat-floating and falling”.

### 3.2. Spectral Amplitude and Spectral Slope

Due to the jet stream area in the troposphere, the buoyancy frequency has a rapidly increasing area. In this interval, the temperature profile has a large disturbance, which is not suitable for spectrum analysis. Therefore, we divide the vertical profile of the rising and falling stages into segments of 1–9 km and 16–24 km. For the flat-floating dataset, we uniformly interpolate the data according to the meridional and zonal directions, and the meridional and zonal step lengths correspond to the average distance between two adjacent points in the X and Y directions, respectively. Two sets of horizontal data in different directions are obtained in this way. It should be noted that, because the horizontal step of the data in the flat-floating stage is different for different profiles, we select 1024 data points here as a segment, divide each group of horizontal drift dataset into several segments and then calculate the meridional wavenumber spectrum and zonal wavenumber spectrum for each piece of data. The characteristics of wavenumber spectrum are characterized by spectrum slope and spectrum amplitude. Considering that the transition from the gravity wave spectrum to the turbulence spectrum reduces the slope of the fit interval, we choose the right boundary of the fitting range as m = 1.0 × 10^−2^ cycle/m, to exclude the influence of turbulence and small-scale noise perturbations. When calculating the vertical wavenumber spectrum for the rising and falling stages, we perform a first-order linear fit to obtain the slope based on the log–log power spectrum in the 9.97 × 10^−4^–1.0 × 10^−2^ cycle/m wavenumber range. In the wavenumber range, the aliasing effect can be ignored. When calculating the horizontal wavenumber spectrum in the flat-floating stage, the fitting interval is selected as 3 × 10^−4^–1.0 × 10^−2^ cycle/m. Considering that the change in the actual spectrum slope causes amplitude swing, the power spectrum density corresponding to the “center of mass” wavenumber [22] is used here as the spectrum amplitude:

(13) $$\bar{\log_{10}k_{n}}=\frac{1}{N_{2}}\sum_{i=j}^{i=k}\log_{10}k_{i}$$

Among them, $k_{j}$ and $k_{k}$ respectively correspond to the left boundary and right boundary of the fitting range, and $N_{2}$ represents the total number of data points. The “center of mass” wavenumber can be written as follows:

(14) $$k_{nc}=10^{\bar{\log_{10}k_{n}}}$$

**Figure 7:**
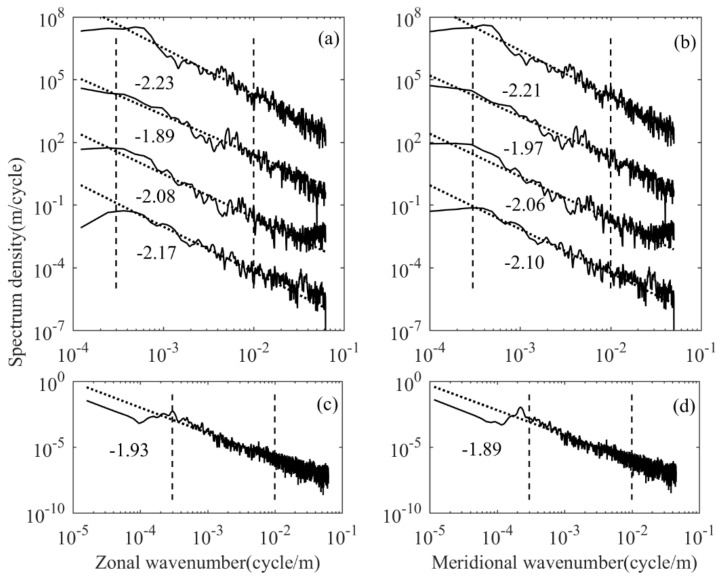
(**a**) The zonal wavenumber spectrum after segmentation, (**b**) the meridional wavenumber spectrum after segmentation, (**c**) the zonal wavenumber spectrum of the entire horizontal part and (**d**) the meridional wavenumber spectrum of the entire horizontal part.

In order to explore the effect of segment length on the spectrum results, the data in the flat-floating stage can be divided into four segments, according to 1024 data bins, and the zonal wavenumber spectrum and the meridional wavenumber spectrum are calculated respectively. At the same time, a spectrum transformation is performed on the data over the entire horizontal section. The results of horizontal wavenumber spectrum are shown in Figure 7. Figure 7a represents the zonal wavenumber spectrum obtained from the four segments of data divided along the Y direction. The fitted spectrum slopes are −2.17, −2.08, −1.89 and −2.23, respectively, and the corresponding spectrum amplitudes are 4.40, 1.67, 1.44 and 1.47 (×10^−4^ cycle/m)^−1^. Figure 7b represents the meridional wavenumber spectrum obtained from four segments of data divided along the X direction. The fitted spectrum slopes are −2.10, −2.06, −1.97 and −2.21, and the corresponding spectrum amplitudes are 5.19, 1.81, 1.52 and 1.63 (×10^−4^ cycle/m)^−1^. The interval between adjacent power spectrum density reflected in the figure is three orders of magnitude. Figure 7c,d shows the zonal and meridional wavenumber spectrum of the entire dataset from the flat-floating stage. It can be seen that the spectrum slope and spectrum amplitude of each segment are not much different, basically around −2. Moreover, the slope and amplitude of the meridional and zonal spectrum of the same segment are very similar. This shows that the segmentation is based on 1024 points. Although the data at different detection times correspond to different horizontal step lengths, this does not affect the results. The meridional and zonal spectrum are highly consistent, showing the isotropy of horizontal temperature disturbances. It should be noted that the increase in spectral amplitude at high wavenumbers is caused by the aliasing effect of noise and spectrum, while the spectral amplitude is significantly reduced at the lowest wavenumbers, due to the filtering effect of removing the linear background.

**Figure 8:**
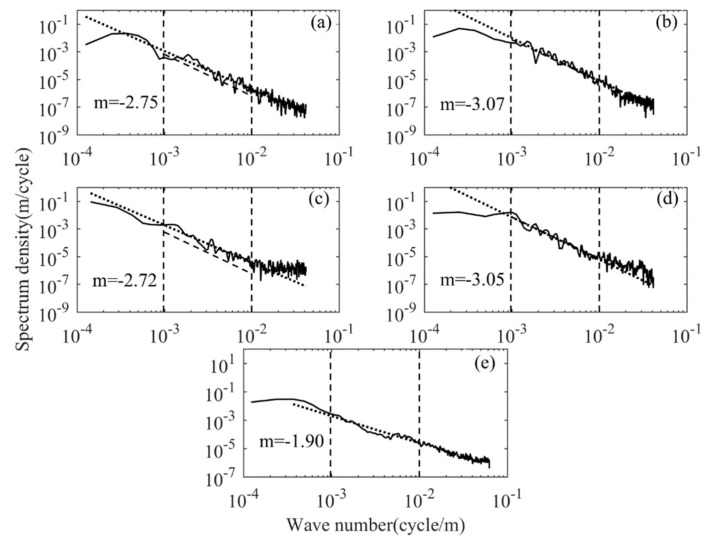
(**a**) Tropospheric vertical wavenumber spectrum in rising stage, (**b**) stratospheric vertical wavenumber spectrum in rising stage, (**c**) tropospheric vertical wavenumber spectrum in falling stage, (**d**) stratospheric vertical wavenumber spectrum in falling stage and (**e**) horizontal wavenumber spectrum in flat-floating stage.

The continuity of the detection data in the three stages of "rising, flat-floating and falling" from RTAFSS allows us to continuously track the variation in the spectrum characteristics during the entire detection period, which has a significant advantage over traditional in situ detection methods. Taking the data of Wuhan on October 23 as an example, Figure 8a,b represents the vertical wavenumber spectrum of the troposphere and stratosphere in the ascending segment; Figure 8c,d represents the vertical wavenumber spectrum of the troposphere and stratosphere in the descending segment; and Figure 8e represents the flat-floating segment. The horizontal wavenumber spectrum is represented by the average spectrum of the four zonal power spectrums. Considering that there are differences in spectrum amplitude and spectrum slope at different moments in the flat-floating stage, the perturbation of a single power spectrum is more obvious. When calculating the horizontal wavenumber spectrum of each sounding, the average wavenumber spectrum is used to reduce the disturbance of the power spectrum and make the spectrum shape smoother; the average zonal wavenumber spectrum is calculated as follows:

(15) $$\log F_{avg}(k_{n})=\frac{1}{N}\sum \log F_{i}(k_{n})$$

where N is the total number of profiles in the horizontal part, i=1,…,N. The obtained zonal average wavenumber spectrum is shown in Figure 8e. The dashed line represents the saturated amplitude spectrum predicted by theoretical spectral model, and the dotted line represents the amplitude spectrum of the best linear fit. The tropospheric and stratospheric spectrum slopes in the rising stage are −2.75 and −3.07; the corresponding spectrum amplitude is 1.62 and 8.62 (×10^−5^ cycle/m) ^−1^; the tropospheric and stratospheric spectrum slopes of the falling stage are −2.72 and −3.05; the corresponding spectral amplitude is 2.72 and 5.98 (×10^−5^ cycle/m^)−1^; the spectrum slope in the flat-floating stage is −1.90; and the corresponding spectral amplitude is 1.19 (×10^−4^ cycle/m)^−1^. The spectrum slope and spectrum amplitude in the stratosphere are higher than those in the troposphere, and the power spectrum of the rising and falling stages are not much different. Figure 8c,e has almost the same power level in the high wavenumber region, which is the same as the case of the second segment of the power spectrum in Figure 7a,b. We suppose this may be due to the influence of noise.

**Figure 9:**
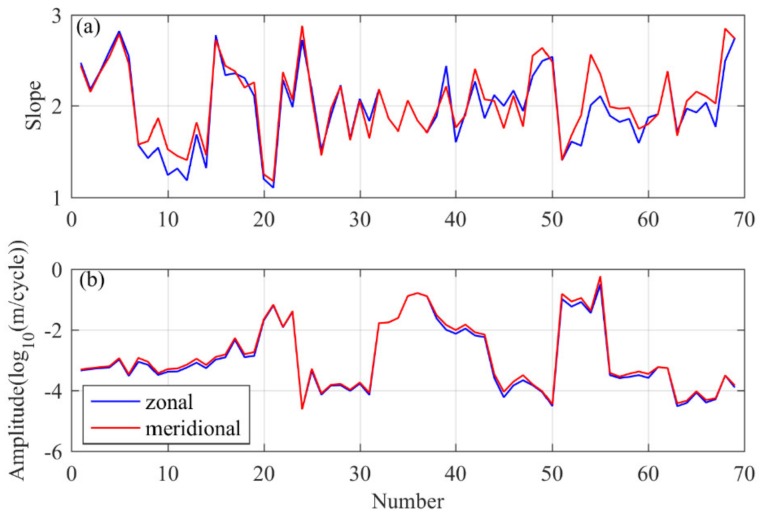
(**a**) Spectrum slopes and (**b**) spectral amplitudes of all data segments obtained according to 1024 data bins segmentation for all selected flat-floating data. The blue curve represents the zonal wavenumber spectrum, and the red curve represents the meridional wavenumber spectrum.

In order to fully understand the horizontal wavenumber spectrum characteristics, we divided a total of 16 sets of horizontal drift data into 69 data segments and calculated the spectrum characteristics of normalized temperature disturbances for each segment. The results of the spectrum slope and spectrum amplitude are shown in Figure 9. The blue curve represents the zonal wavenumber spectrum, and the red curve represents the meridional wavenumber spectrum. The minimum slope is only −1.12, while the maximum slope can reach −3.67 (when describing the magnitude of the spectrum slope, we ignore the negative sign). In general, the spectrum slope basically fluctuates around −2, and the meridional spectrum slope and the zonal spectrum slope are basically the same, although the difference between the spectrum slopes of the individual data segments can reach 0.3, and the average spectrum slope in flat-floating stage is −1.98. For the spectrum amplitude curve, the ordinate is $\log_{10}F_{T}$, where $F_{T}$ is the power spectrum density of the normalized temperature fluctuation. The curves of the zonal and meridional wavenumber spectrum can coincide well, and the trends are completely the same. However, the magnitude of the spectrum amplitude is very different. To investigate the reason, we calculated the temperature variance corresponding to each piece of data. The temperature variance can reflect the dispersion degree of the data. As a result, it is found that a larger spectrum amplitude corresponds to a larger temperature variance. Different temperature variances can correspond to different power levels, but the spectrum shapes are basically the same, and the corresponding spectrum slope differences is also negligible. Stratospheric enhanced gravity wave activity or turbulence generated by gravity wave fragmentation enhances the temperature variance of the horizontal drift data, which is consistent with Cho’s discussion about the effect of horizontal wind speed variance on the horizontal wavenumber spectrum of wind speed fluctuation [17].

**Figure 10:**
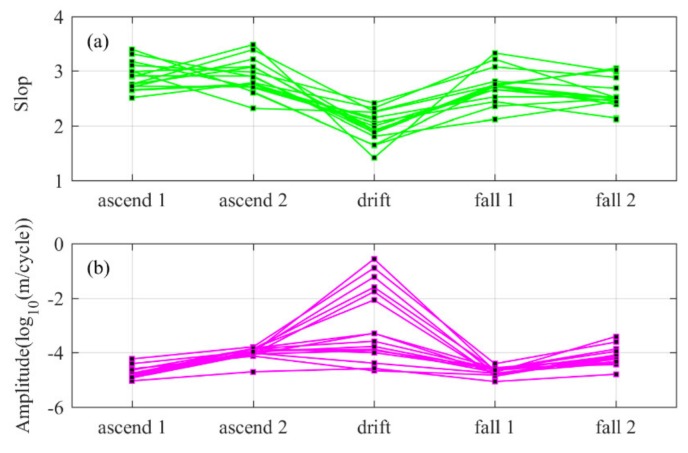
(**a**) Spectrum slope and (**b**) spectrum amplitude of three stages, “rising, flat-floating and falling”, of all detected data. Where ascend 1 and ascend 2 correspond to 1–9 km and 16–24 km of the rising stage; drift corresponds to the flat-floating stage; and fall 1 and fall 2 correspond to 1–9 km, 16–24 km of the falling stage, respectively.

The temperature perturbation spectrum obtained from the three stages of "rising, flat-floating and falling" are combined together, and the results are shown in Figure 10. Figure 10a shows the spectrum slopes at different stages obtained from all sounding data, and Figure 10b shows the corresponding spectrum amplitudes. Here, the spectrum slope of horizontal wavenumber spectrum is the average zonal spectrum of each sounding obtained based on Equation (12), and the average spectrum slope of the 16 datasets is −1.98, which is equal to the average slope of the 69 segments obtained according to 1024 data bins segmentation for all selected flat-floating data. For all data segments of the corresponding height interval in the rising and falling stages, some criteria are used to determine whether the data segment is suitable for extracting the vertical wavenumber spectrum before spectrum transforming: (1) The Brunt–Vaisala frequency is approximately constant, with no significant change; (2) since the rising speed is around 6 m/s and the horizontal wind speed does not exceed about 10 times of the balloon’s rising rate, the distortion in the balloon measurement of the temperature spectrum can be ignored. (3) The wind shear criterion (wind shear > 0.035 s^−1^) is adopted to reject the velocity estimation that is obviously inconsistent with the adjacent points in the spatial sequence. The spectrum slope of the troposphere and stratosphere changes slightly around −3. The change in spectrum slope can be explained by the “wind shifting” theory [1,23], so it is not discussed in detail here. It should be noted that the spectrum slope of the data segments in the falling stage in the stratosphere is lower than that in the rising stage; here, we think that it may be due to the rapid fall in the early stage of the falling stage, and the quality of the temperature data obtained are affected. The spectrum amplitude in the stratosphere is higher than that in the troposphere, regardless of whether the data are from rising or falling stages. If only the gravity waves propagating upward from the ground are considered, they will be more easily absorbed by the “critical layer” caused by the rapids zone near the tropopause when passing through high-altitude jets, so the spectrum amplitude of temperature fluctuation in the stratosphere will decrease [24,25,26]. However, in fact, all 16 sets of data show that the spectrum amplitude in the stratosphere is larger than that in the troposphere, indicating that there are still sources of gravity waves in the stratosphere (above 24 km). Active gravity waves and turbulence generated by the breaking of gravity waves will increase the variance of temperature fluctuation. For the horizontal wavenumber spectrum in the flat-floating stage, the magnitude of the spectrum amplitude covers a large range, the maximum spectrum amplitude is 0.2780 (cycle/m)^−1^ and the minimum spectrum amplitude is 2.12 × 10^−5^ (cycle/m)^−1^. This also shows that the power level of the horizontal wavenumber spectrum is significantly more susceptible by the gravity wave disturbance than the vertical wavenumber spectrum.

### 3.3. Overview and Discussion

Currently, both the dropdown sounding and time-encrypted observation from rocket and aircraft have the problem of high cost, which makes it difficult to maintain the daily operation. As for the RTAFSS, it can implement the delayed and controllable dropdown observation based on the airborne balloon carrier with lower cost and more convenient operation.

Horizontal wavenumber spectrum can be measured by aircraft [15,16,17]. However, aircraft detection is limited by time and space coverage. Due to the limitation of sampling rate and aircraft speed, the obtained spectral structure mainly shows the characteristics of large-scale gravity waves, and the investigation of small-scale turbulence is limited. The quasi-Langrangian movement of balloon observation seems to be more advantageous for studying wave-induced disturbances. Long-duration super pressure balloons can implement the stratosphere flight for a long time (last for weeks), which can characterize the main features of the large- and meso-scale dynamics of this atmospheric region [27,28]. There is no observation in the vertical direction matching the flat-floating stage for both long-duration super pressure balloons and aircraft; however, in our study, through RTAFSS, it can meet controllable high frequency observation, including the three stages, "rising, flat-floating and falling", and characterize the main features of the meso-scale, micro-scale and weather-scale dynamics of this atmospheric region. The spatially complete and temporally continuous spectral structure is obtained, which is essential for further exploration of the evolution of gravity waves and the interaction between the troposphere and stratosphere, but is lacking for other detection methods. Moreover, due to its low cost and ease of operation, it can perform conventional networking observations and improve the current level of observation services.

This paper extends the spectral analysis of the horizontal motion of gravity waves to the middle stratosphere, and we compare the results obtained with previous studies. Schoeberl et al. [29] analyzed kinetic energy and temperature spectra from the horizontal motion, based on long-duration super pressure balloons, and the spectral slope of the temperature spectrum varies from −1.1 to −2.5, with an average of −1.91, which is highly consistent with the distribution of the spectral slope in this paper. This means that, even at different heights, spectral slope of horizontal gravity wave motions should be universal. Cho obtained horizontal wavenumber spectral parameters for wavelength regime 6–60 km and found the slopes generally did not depart greatly from −5/3 [16]. Moreover, in a longer flight segment, a k^−3^ horizontal velocity variance spectrum is displayed at scales longer than about 100 km, indicating that these large-scale fluctuations were likely due to vertical modes rather than inertio-gravity wave [17]. Different from previous studies, the horizontal spectrum displayed in this paper is at scales from tens of meters to several kilometers. The results show some degree of deviation from −5/3 in a much shorter segment, indicating a relatively rapid evolution of gravity waves and turbulence in a small scale. For planet-scale waves, the slope can reach −3, and for smaller-scale waves, the slope will decrease. Since the Kolmogorov scaling (k^−5/3^ slope) is known as the turbulent inertial range, the existence of turbulence can make the slope less negative. Therefore, it is reasonable to believe that such a k^−2^ spectrum represents a transitional subrange for the classic k^−5/3^ to k^−3^ slope [30,31]. As for aircraft detection [32,33], due to the high flight speed, the observed gravity wave scale is large, and the entire detection process takes a short time. However, the time taken in flat-floating stage from RTAFSS is longer, and the disturbances measured in different segments may reflect the characteristics of small-scale gravity wave and Kelvin–Helmholtz wave at different stages, including the intermittent turbulence caused by them, which have caused the obvious differences in the structure of power density spectrum during the entire flat-floating process. It should be noted that, as the height increases, the spectral amplitude first increases to a maximum value and then decreases [23]. Similarly, in the study of [34], the actual spectral amplitude of the stratosphere is still greater than that of the troposphere (for normalized temperature disturbance), which is also similar to ours, indicating that there are still sources of gravity waves in the stratosphere. 

Considering that the increased stability reduced the spectral slopes at longer length scales while decreased stability extended the −5/3 regime to longer scales [35], the spectral structure characteristics of different segments can reflect different atmospheric stability level, while the scale range dominated by the slope of −5/3 (which may be deviated on certain segments) can reflect the scale of turbulent activity. Taking Figure 7 as an example, the power spectrum of four segments is continuous in time and space, and we may get some useful information from the variation of “break point” (the point at which the slope varies markedly from the turbulent scale to the gravity wave scale) [16]. The power spectrum density of the first segment fits the slope well, the deviation is relatively small and no obvious break point can be seen. This indicates that the turbulence activity in this stage is weak. From the second segment to the fourth segment, the break point gradually moves toward a larger length scale (200, 250 and 500 m, respectively), showing that the turbulence activity is enhanced and the gravity wave fragmentation occurs in the last three stages. This prediction is consistent with the fact that the spectrum amplitude is significantly lower in the last three segments than in the first segment. The length scale corresponding to the breaking point of the mean horizontal wave number spectrum is 200 m (Figure 8e). Moreover, the amplitude of vertical wave number spectrum from ascending stage in the stratosphere is higher than that from descending stage, which is consistent with the result of the horizontal wave number spectrum.

As the first application of this detection method in gravity wave analysis, we verified its ideal effect and acquired the spectral characteristics from the middle stratosphere in China, providing reference for more comprehensive gravity wave analysis with more data later. The system can be popularized and used to improve the level of numerical prediction [36]. Meanwhile, the three-stage observation for tracking the continuous weather system [37], as well as the interaction of the stratosphere and troposphere [38] based on it, is worthy of further study.

## 4. Conclusions

The round-trip airborne flat-floating sounding system (RTAFSS) is a new type of meteorological detection system equipped with multifunctional sensor modules, providing continuous, long-term, high-frequency direct observation of the stratosphere in China. Compared with the traditional single-balloon rising detection, the three-stage detection method of "rising, flat-floating and falling" can realize the observation encryption in space and time, which can help improve the understanding of the mechanism and rules of mesoscale weather system and can also promote the development of numerical prediction models. The detection based on this new airborne platform can also provide a new focus for transportation of stratospheric material [39,40,41,42] and disturbance analysis of the upper and middle atmosphere [43,44,45,46]. Therefore, the RTAFSS has a good application prospect.

Based on the 16 datasets containing the complete three stages of “rising, flat-floating and falling” measured by the RTAFSS, the normalized temperature disturbance is used to obtain the vertical wavenumber spectrum from the data in the rising and falling stages. The horizontal wavenumber spectrum (meridional and zonal wavenumber spectrum) is obtained from the data in the flat-floating stage. Due to the irregular trajectory of the horizontal drift section, the zonal and meridional decomposition of the data is performed by calculating the real-time topocentric terrestrial coordinates (X, Y, Z) of the radiosonde. The results show that the spectral slope and spectral amplitude of the meridional and zonal wavenumber spectrum have good consistency, indicating that temperature disturbances affected by gravity waves and turbulence are isotropic in the horizontal direction of the stratosphere. For the vertical wavenumber spectrum, the values of the spectral slope in the troposphere and stratosphere deviate from the “−3” theoretical spectrum; this is because changes in the background wind field will simultaneously produce systemic variations in velocity variance and wavenumber, which will affect the shape of the power spectrum. For the horizontal wavenumber spectrum, the spectral slope is basically around −2, and the spectral amplitude is obviously affected by the temperature variance. The enhanced temperature variance reflects the strengthening of the turbulence activity caused by the gravity wave and the breaking of the gravity wave.

By extracting the characteristics of the gravity wave spectrum from the dataset of the RTAFSS, we verified the feasibility of the system in the application of gravity wave spectrum analysis. For the first time, we obtained the horizontal wavenumber in China through an airborne platform, which provides a new way for the study of the horizontal wavenumber spectrum of gravity waves. It is the first time in the world to use a controllable sounding that includes the three stages of "rising, flat-floating and falling". This article is an experimental investigation of the application of this type of data in the analysis of gravity wave spectrum. It provides an important reference for more comprehensive analysis of the characteristics of the gravity wave spectrum after RTAFSS networking observations. Considering the superiority of the data itself, it can be possible to explore the influence of the horizontal motion of stratospheric gravity waves on the troposphere, which will be our further study.

## Figures and Tables

**Table 1:** Specific details of the 16 released balloons.

Number	Release Date	Ascend Time /s	Ascending Speed (m/s)	Height Range /m	Drift Time/s	Vertical Speed/ (m/s)	Height Range /m	Fall Time /s	Falling Speed/ (m/s)	Height Range /m
1 Wuhan	2018.10.22 19:30:59	0–4898	5.86	29–28,900	4900–23,340	0.08	28,862–30,391	23,342–27,800	−6.45	30,253–654
2 Wuhan	2018.10.23 07:44:50	0–5038	5.28	34–26,645	5040–22,508	0.02	25,500–27,183	22,510–27,232	−5.39	27,015–1579
3 Wuhan	2018.10.30 07:33:17	0–4296	6.01	29–25,846	4298–23,202	0.05	23,200–26,923	23,204–27,726	−5.70	26,739–658
4 Wuhan	2018.11.01 19:27:23	0–4446	5.97	133–27,120	4448–23,558	0.16	27,042–30,116	23,560–28,098	−6.44	30,019–235
5 Anqing	2018.10.17 07:20:59	0–5274	5.21	68–27,579	5276–34,782	–0.04	25,560–27,880	34,784–44,520	−1.93	26,511–7688
6 Anqing	2018.10.19 19:30:59	0–5788	4.90	86–28,450	5790–34,510	−0.09	25,200–28,560	34,512–46,932	−1.96	25,763–1236
7 Anqing	2018.10.22 19:30:59	0–6902	4.05	62–28,331	6904–24,218	0.03	28,292–28,932	24,220–28,446	−6.30	28,721–1161
8 Anqing	2018.11.10 19:23:02	0–5028	4.32	70–22,023	5030–23,818	0.29	21,894–27,496	23,820–28,220	−6.01	27,318–868
9 Yichang	2018.10.18 19:20:06	0–4672	5.74	256–27,073	4674–24,000	−0.03	26,700–27,194	24,002–28,604	−5.16	26,573–1261
10 Yichang	2018.10.22 19:19:08	0–6890	4.30	256–30,121	6892–27,020	0.03	30,044–30,639	27,022–32,214	−5.80	30,419–299
11 Yichang	2018.10.25 07:19:30	0–4772	5.77	259–27825	4774–24022	−0.04	25,100–27,975	24,024–28,522	−4.99	27,061–1220
12 Yichang	2018.11.13 19:18:42	0–5028	5.47	357–25790	5030–25818	0.12	25,800–28,070	23,820–28,302	−6.24	28,014–1121
13 Ganzhou	2018.10.25 07:27:22	0–4674	5.54	137–26031	4676–23556	0.01	24,017–26,436	23,558–27,310	−6.33	26,306–2508
14 Ganzhou	2018.10.31 19:21:15	0–6198	4.69	156–29326	6200–23856	−0.02	29,100–29,600	23,858–28,704	−5.69	29,045–1315
15 Ganzhou	2018.11.06 07:35:23	0–4170	5.75	238–24,312	4172–23,076	−0.01	23,030–24,500	23,078–27,262	−5.43	24,195–1560
16 Ganzhou	2018.11.09 19:23:31	0–6008	4.61	192–28,995	6010–23,788	−0.01	28,820–29,200	23,790–28,154	−6.08	28,750–1930
